# Bioinformatic analysis reveals lysosome-related biomarkers and molecular subtypes in preeclampsia: novel insights into the pathogenesis of preeclampsia

**DOI:** 10.3389/fgene.2023.1228110

**Published:** 2023-07-28

**Authors:** Yao Chen, Miao Liu, Yonghong Wang

**Affiliations:** Department of Obstetrics, The First People’s Hospital of Chenzhou, Chenzhou, China

**Keywords:** preeclampsia, bioinformatics, lysosome, GSVA, GSEA, immune cell infiltration

## Abstract

**Background:** The process of lysosomal biogenesis and exocytosis in preeclamptic placentae plays a role in causing maternal endothelial dysfunction. However, the specific lysosome-associated markers relevant to preeclampsia (PE) are not well-defined. Our objective is to discover new biomarkers and molecular subtypes associated with lysosomes that could improve the diagnosis and treatment of PE.

**Methods:** We obtained four microarray datasets related to PE from the Gene Expression Omnibus (GEO) database. The limma package was utilized to identify genes that were differentially expressed between individuals with the disease and healthy controls. The logistic regression analysis was used to identify core diagnostic biomarkers, which were subsequently validated by independent datasets and clinical samples. Additionally, a consensus clustering method was utilized to distinguish between different subtypes of PE. Following this, functional enrichment analysis, GSEA, GSVA, and immune cell infiltration were conducted to compare the two subtypes and identify any differences in their functional characteristics and immune cell composition.

**Results:** We identified 16 PE-specific lysosome-related genes. Through regression analysis, two genes, *GNPTG* and *CTSC*, were identified and subsequently validated in the external validation cohort GSE60438 and through qRT-PCR experiment. A nomogram model for the diagnosis of PE was developed and evaluated using these two genes. The model had a remarkably high predictive power (AUC values of the training set, validation set, and clinical samples were 0.897, 0.788, and 0.979, respectively). Additionally, two different molecular subtypes (C1 and C2) were identified, and we found notable variations in the levels of immune cells present in the two subtypes.

**Conclusion:** Our results not only offered a classification system but also identified novel diagnostic biomarkers for PE patients. Our findings offered an additional understanding of how to categorize PE patients and also highlighted potential avenues for creating treatments for individuals with PE.

## Introduction

Preeclampsia (PE) is a pregnancy disorder that usually occurs after 20 weeks of pregnancy, and is characterized by high proteinuria and blood pressure (Ma’ayeh and Costantine, 2020; Ramos et al., 2017). This condition is linked to significant maternal morbidity and mortality, as well as fetal growth restriction, iatrogenic premature delivery, and organ dysfunction (Mol et al., 2016). Moreover, infants born to mothers with PE are at risk for adverse neurological outcomes, such as schizophrenia, autism, epilepsy, and intellectual disability (Byrne et al., 2007; Wu et al., 2008; Griffith et al., 2011; Walker et al., 2015; Ursini et al., 2018). Currently, there are limited clinical interventions available that are effective in treating PE. Even after delivery, both infant and mother are still susceptible to chronic renal or hypertension later in life (Irgens et al., 2001; Skjaerven et al., 2012). Therefore, identifying pregnant women at high risk of developing PE or severe PE promptly is crucial to prevent adverse pregnancy outcomes. Currently, PE is diagnosed based on proteinuria and hypertension. However, this approach lacks specificity and sensitivity, resulting in a poor prognosis for both the mother and fetus (Zhang et al., 2001). Therefore, it is imperative to identify novel diagnostic biomarkers that can be used for monitoring and diagnosis of PE.

The development of PE is a multifaceted process that involves various mechanisms such as inflammatory processes, immune imbalance, and oxidative stress (Taysi et al., 2019; Wang et al., 2022; Deer et al., 2023). A previous study has highlighted the significance of autophagy in vascular remodeling and trophoblast invasion (Saito and Nakashima, 2014). Lysosomes are crucial organelles within cells that serve as both degradation centers and signaling hubs. They play integral roles in maintaining cellular homeostasis, promoting development, and influencing the aging process (Yang and Wang, 2021). Lysosomes are also known to play a crucial role in autophagy and endocytosis and can originate from pre-existing autolysosomes or endolysosomes (Mahapatra et al., 2021). They are involved in different types of autophagy, such as macroautophagy and microautophagy, as well as various cell death pathways including autophagic cell death, apoptotic cell death, and lysosomal cell death (Radulovic et al., 2018; Wang et al., 2018). The pathogenesis and treatment of PE involve immune and inflammatory reactions, and lysosomes with immunogenicity may implicate the pathophysiological process of PE (Visser et al., 2007; Watts, 2022). Therefore, identifying lysosome-related genes (LRs) is crucial for early diagnosis of PE and may aid in early intervention for affected patients. However, the exact mechanism of lysosomes in the PE is still unclear.

**FIGURE 1:**
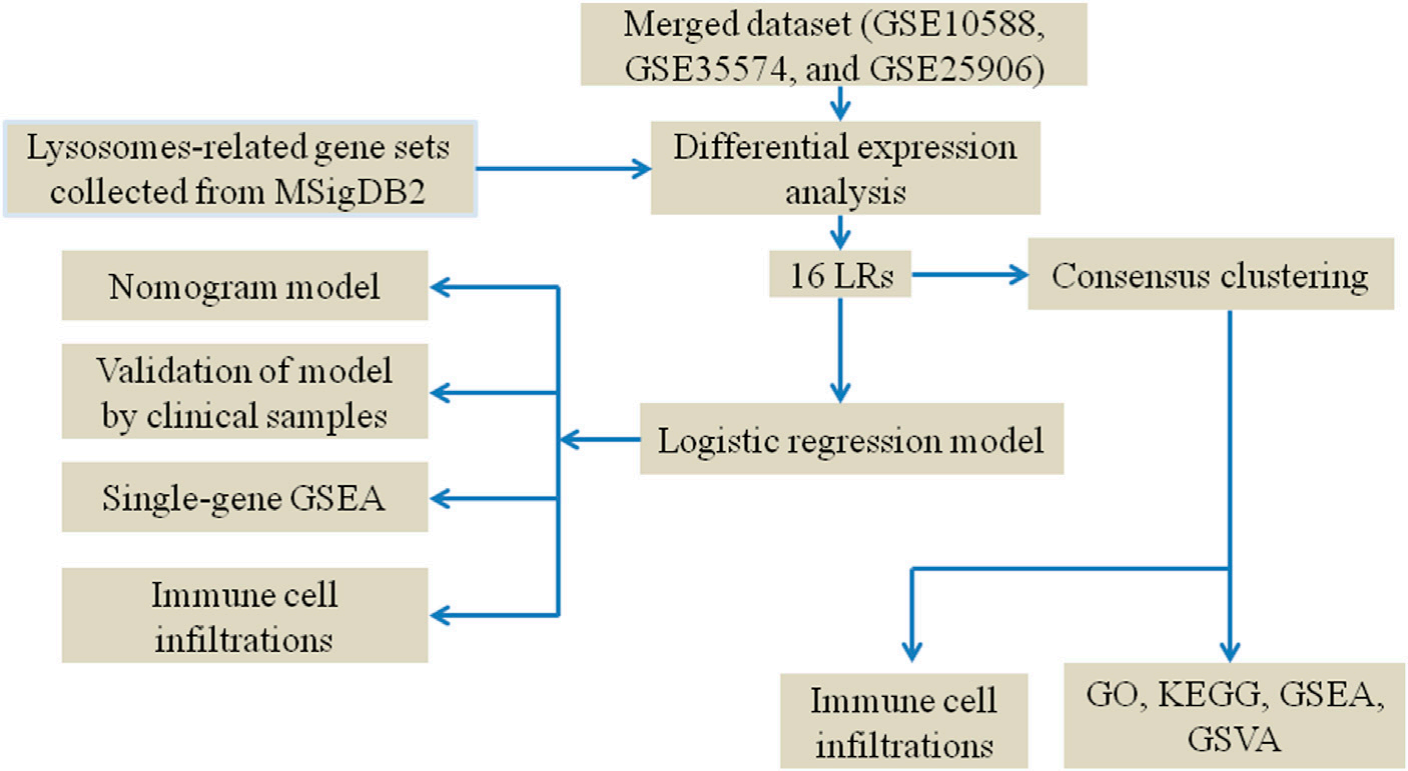
The flow chart of the process of the analysis.

The pathophysiology and etiology of PE are currently not well understood and are being extensively researched. It is widely recognized that the placenta has a significant impact on the development of PE, and research focused on the changes in placental pathophysiology is particularly valuable (van der Merwe et al., 2010; Travaglino et al., 2019; Kim et al., 2021). In the present study, we utilized a data-mining approach to identify the differentially expressed LRs in placenta samples from PE and healthy controls (HC). We employed logistic regression analysis to identify potential genes that serve as risk signatures for predicting the likelihood of PE occurrence. We then created a nomogram model using these diagnostic genes. Furthermore, we examined the relationship between immune infiltration and the diagnostic genes and performed a GSEA analysis on them. Furthermore, to identify subtypes associated with lysosome phenotypes in PE samples, consensus clustering was performed. A theoretical basis for understanding the lysosomes associated with PE development will be provided through our findings. The workflow of this study is illustrated in Figure 1.

## Materials and methods

### Collection of the datasets and data processing

Four microarray datasets (GSE10588, GSE35574, GSE25906, and GSE60438) related to PE were obtained from the Gene Expression Omnibus (GEO) database (http://www.ncbi.nlm.nih.gov/geo/). Table 1 presents the relevant information for these datasets. To remove any batch effects, the “sva” package in R was used to merge three of the datasets (GSE10588, GSE35574, and GSE25906). To categorize the samples, the merged dataset was divided into HC (*n* = 103) and PE (*n* = 59) groups. The merged dataset was then used as a training set, while GSE60438 was used as a validation set. Lysosome-related gene sets were collected from the Molecular Signatures Database. After removing duplicates, 180 LRs were obtained.

**TABLE 1:** Information of the microarray datasets in the present study.

GEO id	Platform	HC	PE	Application
GSE10588	GPL2986	26	17	Training
GSE35574	GPL6102	40	19	Training
GSE25906	GPL6102	37	23	Training
GSE60438	GPL10558	42	35	Validation

### Identification of differentially expressed LRs

We utilized the “limma” package in R to screen for differentially expressed LRs between HC and PE samples, applying a threshold of p.adjust <0.05 and |log fold change (FC)| ≥ 0.5. To create the heatmap, we used the “pheatmap” package, and for the volcano plot, we used the “ggplot2” package.

### Identification of diagnostic markers and construction of nomogram model

To identify important genes associated with PE, logistic regression analysis was used to screen for these genes from the differentially expressed LRs. The accuracy of the diagnostic model was evaluated using receiver operating characteristic (ROC) curve analysis, both in the merged dataset and in the GSE60438 dataset. To investigate the specific role of important LRs in diagnosing PE, we utilized the “rms” package to develop a nomogram model for predicting the incidence of PE. The model’s accuracy was evaluated using calibration curves and decision curve analysis (DCA) (Iasonos et al., 2008).

### Single-gene gene set enrichment analysis (GSEA)

For single-gene GSEA, we utilized the “clusterProfiler” and “enrichplot” packages to classify samples into high and low expression groups using the median values of gene expression levels. We obtained the subset “c2.cp.kegg.v7.4.symbols.gmt” from the Molecular Signatures Database to investigate molecular mechanisms of genes based on phenotypic grouping.

### Analysis of immune cell infiltration

The xCell algorithm was utilized in this study to compare the enrichment scores of infiltrating immune cells between PE and HC groups. The algorithm is a novel method for assessing the relative fraction of immune cells based on gene signatures (Aran, 2020). The correlation between the immune cells and the signature genes was analyzed using the “ggplot2” package and visualized in a lollipop diagram.

### Identification of molecular subtypes

Unsupervised clustering analysis of PE patients was performed using ConsensusClusterPlus based on differentially expressed LRs. This helped to classify the PE samples into several subtypes. Subsequently, we used the “limma” package to screen differentially expressed genes (DEGs) between the two subtypes. To investigate the distinct signaling pathways and possible roles of DEGs, we performed Kyoto encyclopedia of genes and genomes (KEGG) and gene ontology (GO) enrichment analyses using the R package “clusterProfiler”. A q-value of less than 0.05 was deemed significant.

### Gene set variation analysis (GSVA)

In this study, we obtained the “c5.go.symbols” subsets from the MSigDB database to examine variations in biological pathways. To analyze the differences in pathways and biological functions among various subtypes, we employed the “Limma” and “GSVA” packages.

### Validation of the bioinformatics results

Placenta specimens from 12 HC to 12 PE patients were collected from the Department of Obstetrics, The First Peple’s Hospital of Chenzhou. The study obtained written informed consent from all participants and was approved by the Ethics Committee of The First People’s Hospital of Chenzhou. Tissue total RNA was extracted using TRIzol reagent (Invitrogen; Thermo Fisher) following the manufacturer’s protocols. Additionally, the RNA was quantified spectrophotometrically at an absorbance of 260 nm, and its purity was estimated by calculating the absorbance ratio at 260/280 nm. In RNA samples, the absorbance ratios (260/280 nm) should ideally range from 1.8 to 2.0. Approximately 2 µg total RNA was converted into cDNA using cDNA synthesis kits (Invitrogen; Thermo Fisher). Subsequently, quantitative real-time polymerase chain reaction (qRT-PCR) analysis was performed on a CFX96™ real-time PCR system (Bio-Rad Laboratories, Inc). GAPDH was used as the reference mRNA. The 2^−ΔΔCt^ method was used to evaluate mRNA gene expression relative to GAPDH expression. The list of primers used can be found in Table 2.

**TABLE 2:** Sequences of primers used quantitative real-time PCR (qRT-PCR).

Gene	Forward primer (5′to 3′)	Reverse primer (5′to 3′)
GNPTG	TGG​CCG​ATG​AGC​TGA​TCA​C	CCT​CAA​AAA​GTG​TCC​TCA​GCA​A
CTSC	CGA​TGT​CAA​CTG​CTC​GGT​TA	GAT​GGT​GAA​ATG​GCC​AGA​AT
GAPDH	CTT​TGT​CAA​GCT​CAT​TTC​CTG​G	TCT​TCC​TCT​TGT​GCT​CTT​GC

## Results

### Identification of differentially expressed LRs in PE

**FIGURE 2:**
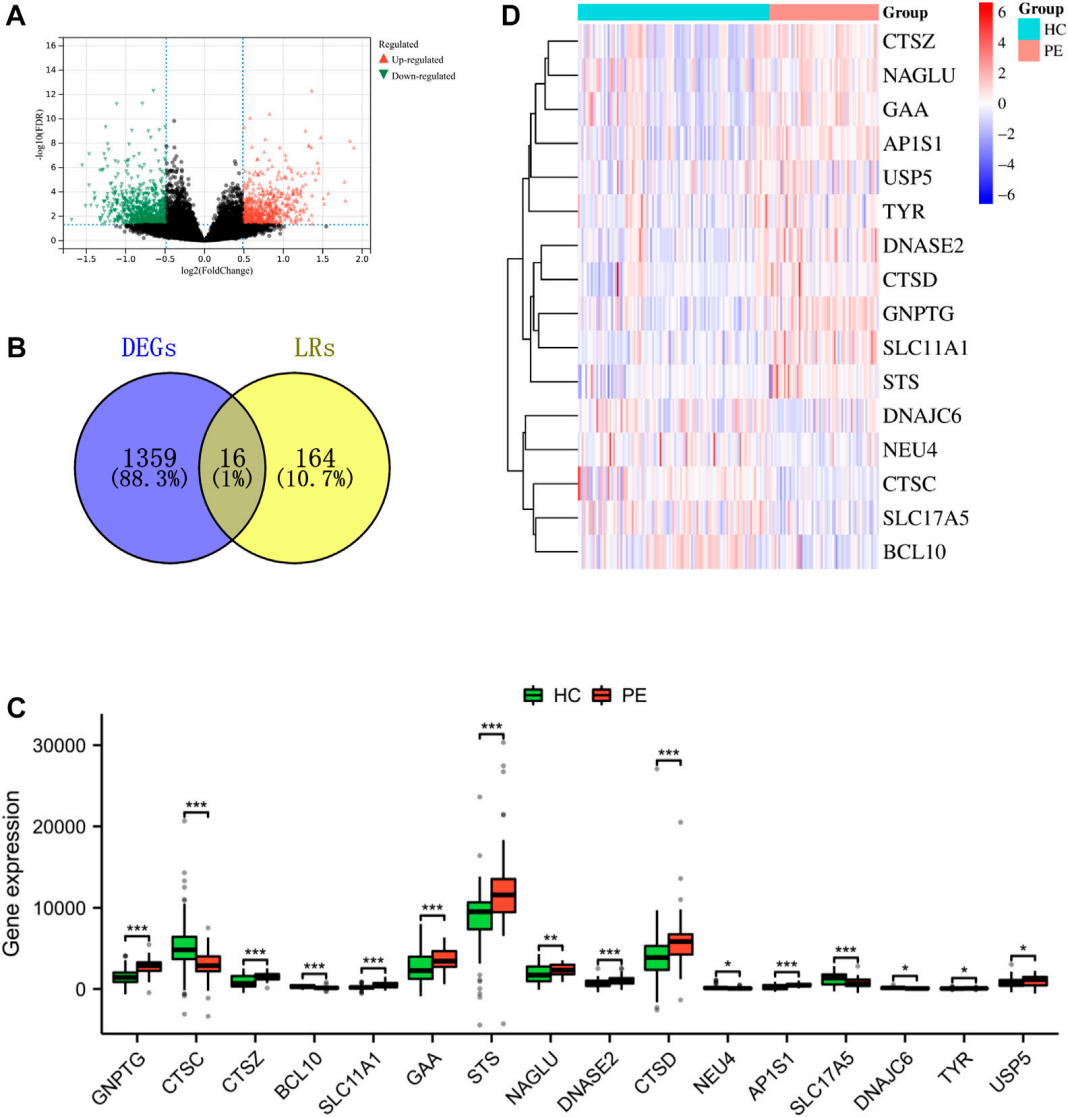
Identification of LRs that are differentially expressed in the merged dataset. **(A)** The volcano plot of DEGs in a combined dataset. **(B)** Venn diagram of differentially expressed LRs resulted from the intersection of LRs and DEGs. Boxplot **(C)** and heatmap **(D)** depicted the differential expression of LRs. *p < 0.05, **p < 0.01, ***p < 0.001.

The integrated dataset used in this study included 59 PE samples and 103 HC samples, which were integrated after removing batch effects from three GEO datasets (GSE10588, GSE35574, and GSE25906). After conducting a differential analysis between PE and HC groups, 1375 differentially expressed genes were identified. Among them, 627 genes were upregulated, while 748 genes were downregulated (Figure 2A and Supplementary Table S1). The genes obtained from the DEGs and LRs were intersected, and 16 differentially expressed LRs were obtained (Figure 2B). To analyze the differential expression of differentially expressed LRs between the PE samples and HC samples, a boxplot, and heatmap were generated to display the expression levels of these genes (Figures 2C, D). The N-Acetylglucosamine-1-Phosphate Transferase Subunit Gamma (*GNPTG*), Cathepsin Z (*CTSZ*), Solute Carrier Family 11 Member 1 (*SLC11A1*), Alpha Glucosidase (*GAA*), Steroid Sulfatase (*STS*), N-Acetyl-Alpha-Glucosaminidase (*NAGLU*), Deoxyribonuclease 2 (*DNASE2*), Cathepsin D (*CTSD*), Adaptor Related Protein Complex 1 Subunit Sigma 1 (*AP1S1*), Tyrosinase (*TYR*), and Ubiquitin Specific Peptidase 5 (*USP5*) genes were found to be upregulated in the PE group compared to the HC group, while Cathepsin C (*CTSC*), B cell lymphoma/leukemia 10 (*BCL10*), Neuraminidase 4 (*NEU4*), Solute Carrier Family 17 Member 5 (*SLC17A5*), and DnaJ Heat Shock Protein Family (Hsp40) Member C6 (*DNAJC6*) genes were downregulated in PE group compared to HC group (*p* < 0.05). In addition, we analysed the gene expression levels of 16 LRs using the GSE60438 dataset. As shown in Supplementary Figure S1, except for *SLC11A1*, *NEU4*, *DNAJC6*, and *TYR*, the expression levels of the other 12 LRs were consistent with the trend of the integrated dataset.

### The logistic analysis identifies diagnostic markers for PE patients

**FIGURE 3:**
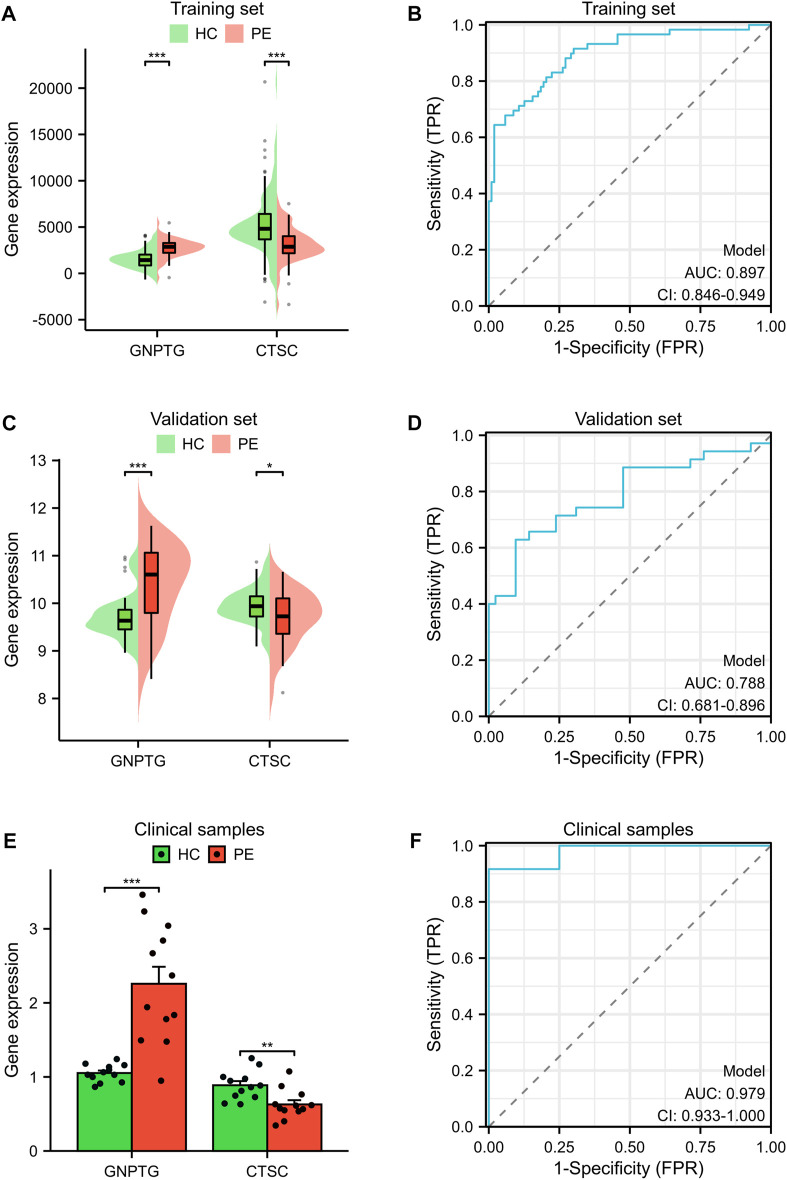
The key diagnostic biomarkers validated by validation set and clinical samples. Gene expression levels of *GNPTG* and *CTSG* in the training set **(A)**, validation set **(C)**, and clinical samples **(E)**. Receiver operating characteristic curves exhibited the diagnostic value of the logistic regression model in the training set **(B)**, validation set **(D)**, and clinical samples **(F)**. *p < 0.05, **p < 0.01, ***p < 0.001.

A logistic regression analysis was conducted using 16 LRs, resulting in the identification of two hub genes (*GNPTG* and *CTSC*) that exhibited *p* values <0.05 (Table 3). These genes were found to be essential for the diagnostic model. In the training set (Figure 3A) and validation set (Figure 3C), the *GNPTG* gene was significantly upregulated in the PE group compared to the HC group, while *CTSC* was significantly downregulated in the PE group (*p* < 0.05). The model achieved an area under the curve (AUC) value of 0.897 on the training set (Figure 3B) and 0.788 on the external validation set (Figure 3D) based on ROC analysis. To validate the results of the bioinformatics analysis, clinical samples were collected. The results were consistent with those described above (Figures 3E, F).

**TABLE 3:** The results of univariate and multivariate logistic regression.

Characteristics	Total(N)	OR (95% CI) univariate analysis	*p*-Value univariate analysis	OR (95% CI) multivariate analysis	*p*-Value multivariate analysis
**GNPTG**	162	1.001 (1.001–1.002)	**< 0.001**	1.003 (1.002–1.005)	**< 0.001**
**CTSC**	162	1.000 (1.000–1.000)	**< 0.001**	0.999 (0.998–0.999)	**< 0.001**
CTSZ	162	1.001 (1.001–1.002)	<0.001	1.004 (1.001–1.007)	0.14
BCL10	162	0.996 (0.995–0.998)	<0.001	1.010 (1.001–1.019)	0.31
SLC11A1	162	1.004 (1.002–1.005)	<0.001	1.001 (0.998–1.005)	0.385
GAA	162	1.000 (1.000–1.001)	<0.001	0.999 (0.998–1.000)	0.05
STS	162	1.000 (1.000–1.000)	<0.001	1.000 (1.000–1.000)	0.166
NAGLU	162	1.001 (1.000–1.001)	0.002	1.002 (1.000–1.003)	0.07
DNASE2	162	1.001 (1.001–1.002)	<0.001	0.997 (0.995–0.999)	0.062
CTSD	162	1.000 (1.000–1.000)	0.004	1.000 (1.000–1.001)	0.140
NEU4	162	0.997 (0.995–0.999)	0.012	0.996 (0.989–1.002)	0.215
AP1S1	162	1.003 (1.002–1.004)	<0.001	1.003 (1.000–1.006)	0.072
SLC17A5	162	0.999 (0.998–0.999)	<0.001	0.999 (0.997–1.000)	0.058
DNAJC6	162	0.996 (0.993–0.999)	0.003	0.987 (0.978–0.995)	0.31
TYR	162	1.003 (0.998–1.009)	0.224	N/A	N/A
USP5	162	1.001 (1.000–1.001)	0.060	1.001 (0.999–1.002)	0.269

Bold values represents a statistically significant difference.

### Establishment of the nomogram model

**FIGURE 4:**
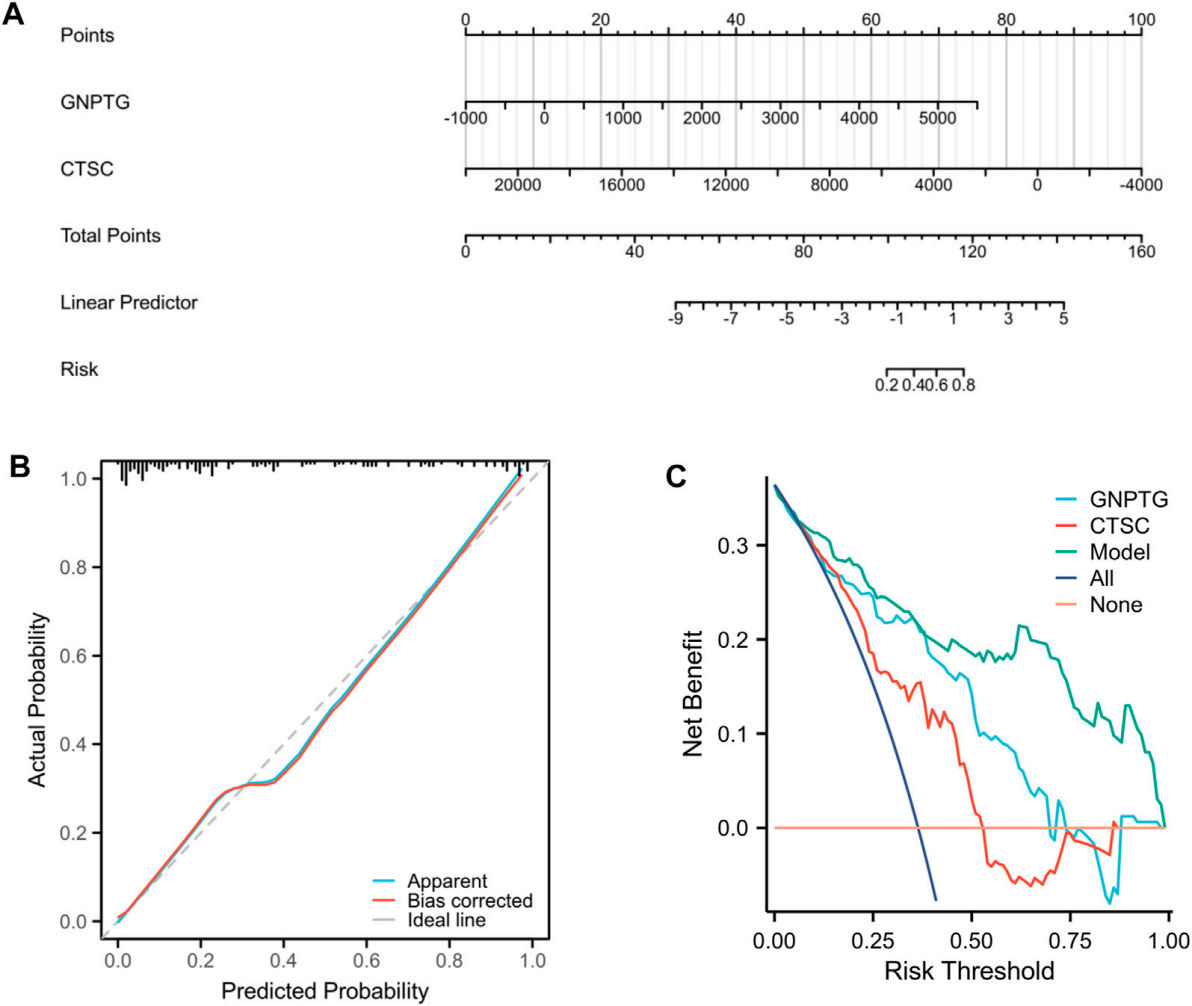
Nomogram model. **(A)** Nomogram of two trait genes and predicted risk score of PE patients. **(B)** The nomogram model was evaluated and found to have better agreement with the ideal model in terms of diagnostics. **(C)** DCA is based on the nomogram model.

A nomogram model was developed using the projected risk score and two trait genes of the patient to predict the occurrence of PE (Figure 4A). The calibration curves showed that the predictions made by the nomogram model were almost identical to those made by the ideal model (Figure 4B). The decision curve analysis (DCA) demonstrated that the utilization of the nomogram model may be beneficial for patients with PE as the bottle green line (model) consistently outperformed the bottle blue (all) and orange-red (none) lines from 0 to 1 (Figure 4C).

### Studying specific signaling mechanisms associated with trait genes

**FIGURE 5:**
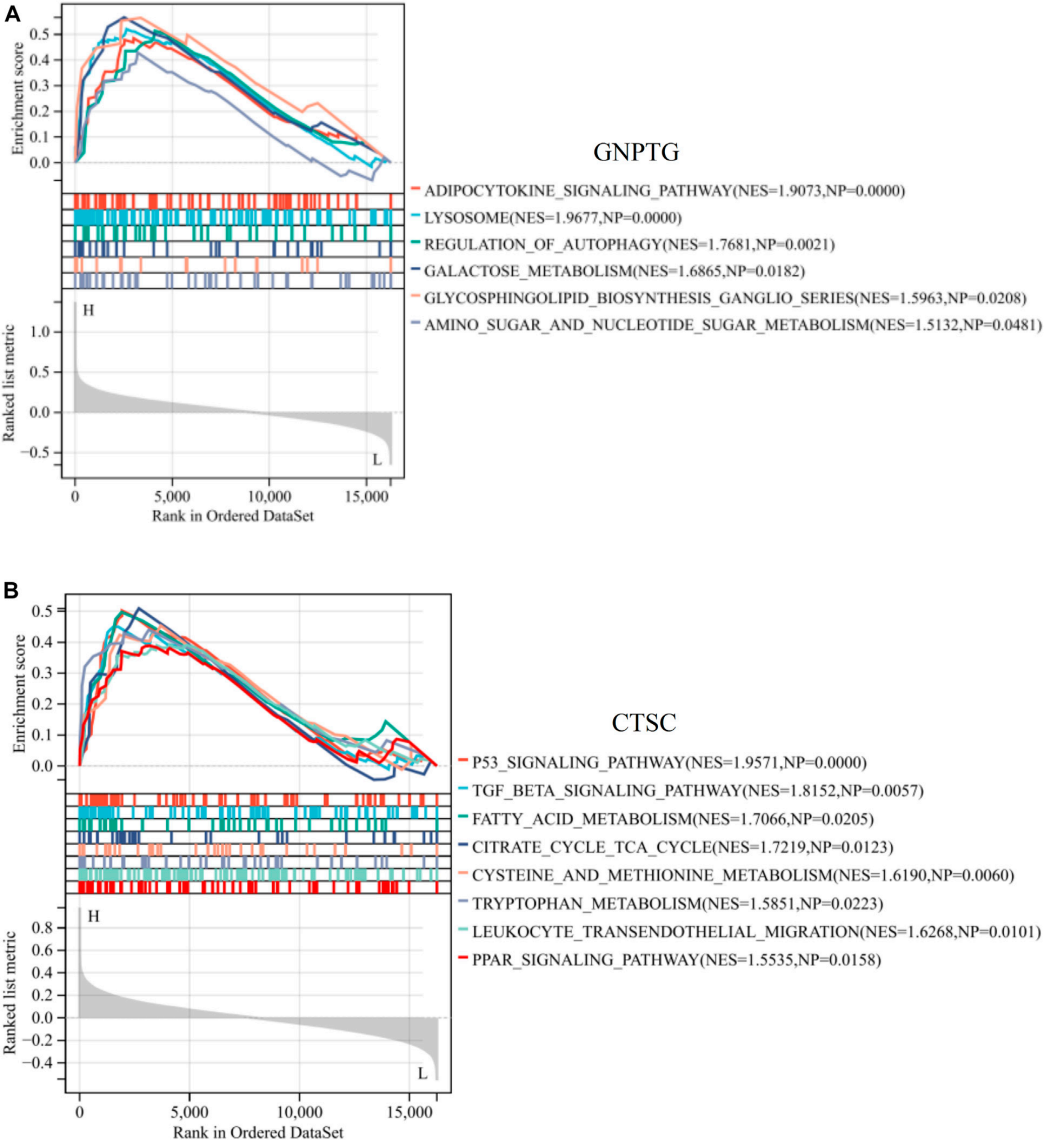
GSEA of trait genes in PE. Single-gene GSEA of *GNPTG* **(A)** and *CTSC* **(B)**. Significantly enriched gene sets were those with a *p*-value <0.05.

To investigate the potential functions regulated by *GNPTG* and *CTSC* in PE, a single-gene GSEA analysis was conducted. The results revealed that lysosome (NES = 1.96, *p* = 0), adipocytokine signaling pathway (NES = 1.90, *p* = 0), galactose metabolism (NES = 1.68, *p* = 0.01), regulation of autophagy (NES = 1.76, *p* = 0.002), glycosphingolipid biosynthesis ganglio series (NES = 1.59, *p* = 0.02), and amino sugar and nucleotide sugar metabolism (NES = 1.51, *p* = 0.04) were enriched in the GNPTG-high subgroup (Figure 5A); P53 signaling pathway (NES = 1.95, *p* = 0), TGF BETA signaling pathway (NES = 1.81, *p* = 0.005), leukocyte transendothelial migration (NES = 1.62, *p* = 0.01), and fatty acid metabolism (NES = 1.70, *p* = 0.02) were enriched in the CTSC-high subgroup (Figure 5B).

### The landscape of the immune cell infiltrates in PE

**FIGURE 6:**
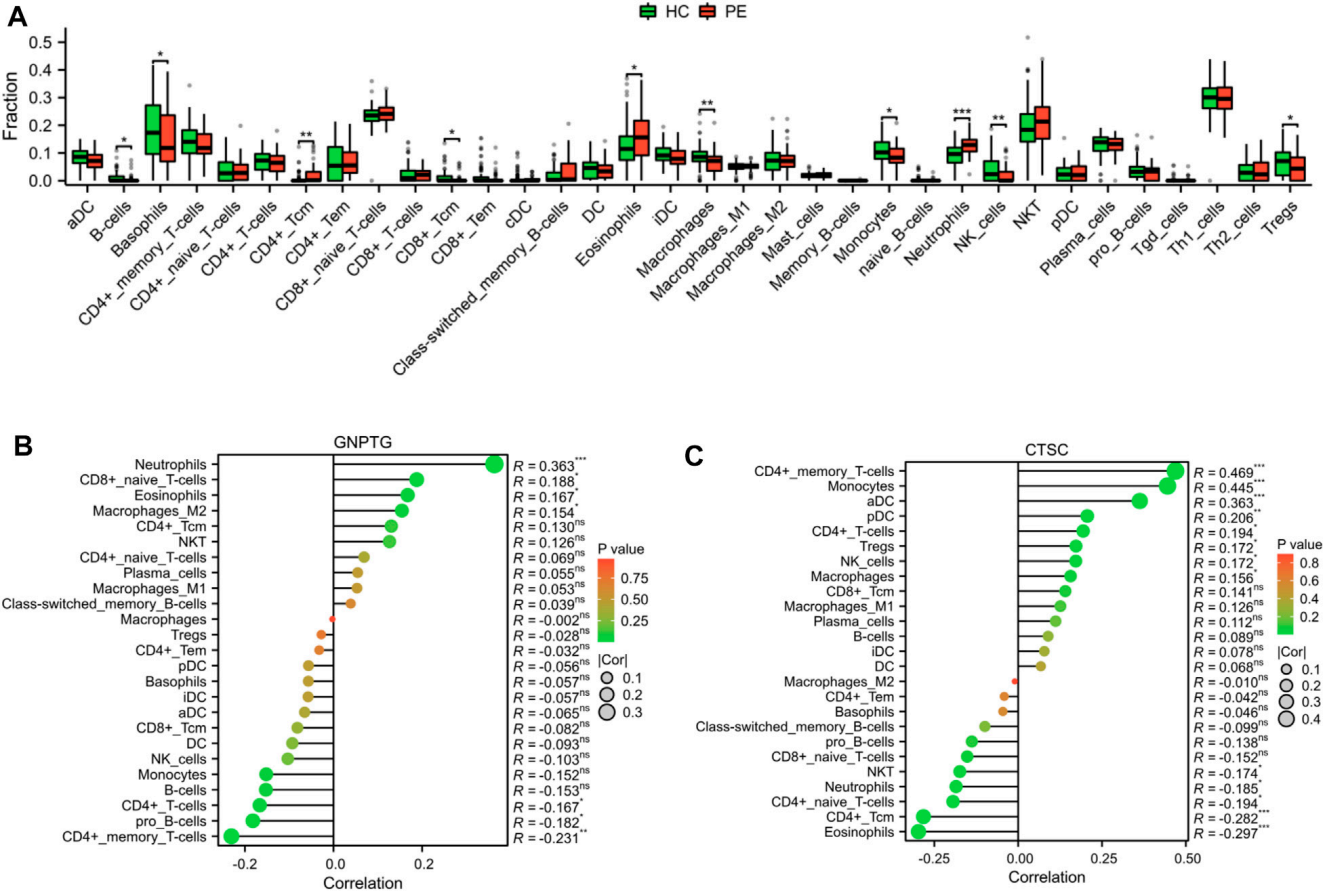
Landscape of the immune cell infiltrates in PE. **(A)** Boxplot depicted the differential infiltration of the immune cells. Correlation analysis of the *GNPTG* **(B)** and *CTSC* **(C)** gene expression with immune infiltration. *p < 0.05, **p < 0.01, ***p < 0.001.

As demonstrated in Figure 6A, we utilized xCell to measure the proportion of immune cell types. The findings revealed that the proportion of CD4^+^ Tcm (*p* < 0.01), eosinophils (*p* < 0.05), and neutrophils (*p* < 0.001) was notably greater in the PE group compared to the HC group; however, the proportion of B cells (*p* < 0.05), basophils (*p* < 0.05), CD8^+^ Tcm (*p* < 0.05), macrophages (*p* < 0.01), monocytes (*p* < 0.05), NK cells (*p* < 0.01), and Tregs (*p* < 0.05) was notably lower in the PE group compared to the HC group. In addition, a correlation analysis was conducted to examine the relationship between trait gene expression and levels of immune cell infiltration. The findings indicated that the expression of these genes was significantly correlated with infiltration levels in various immune cells, including neutrophils, eosinophils, CD4^+^ memory T cells, and CD4^+^ T cells, indicating that these diagnostic genes may play a role in immune regulation in the development of PE (Figures 6B, C).

### Identification of lysosome-related subtypes in PE

**FIGURE 7:**
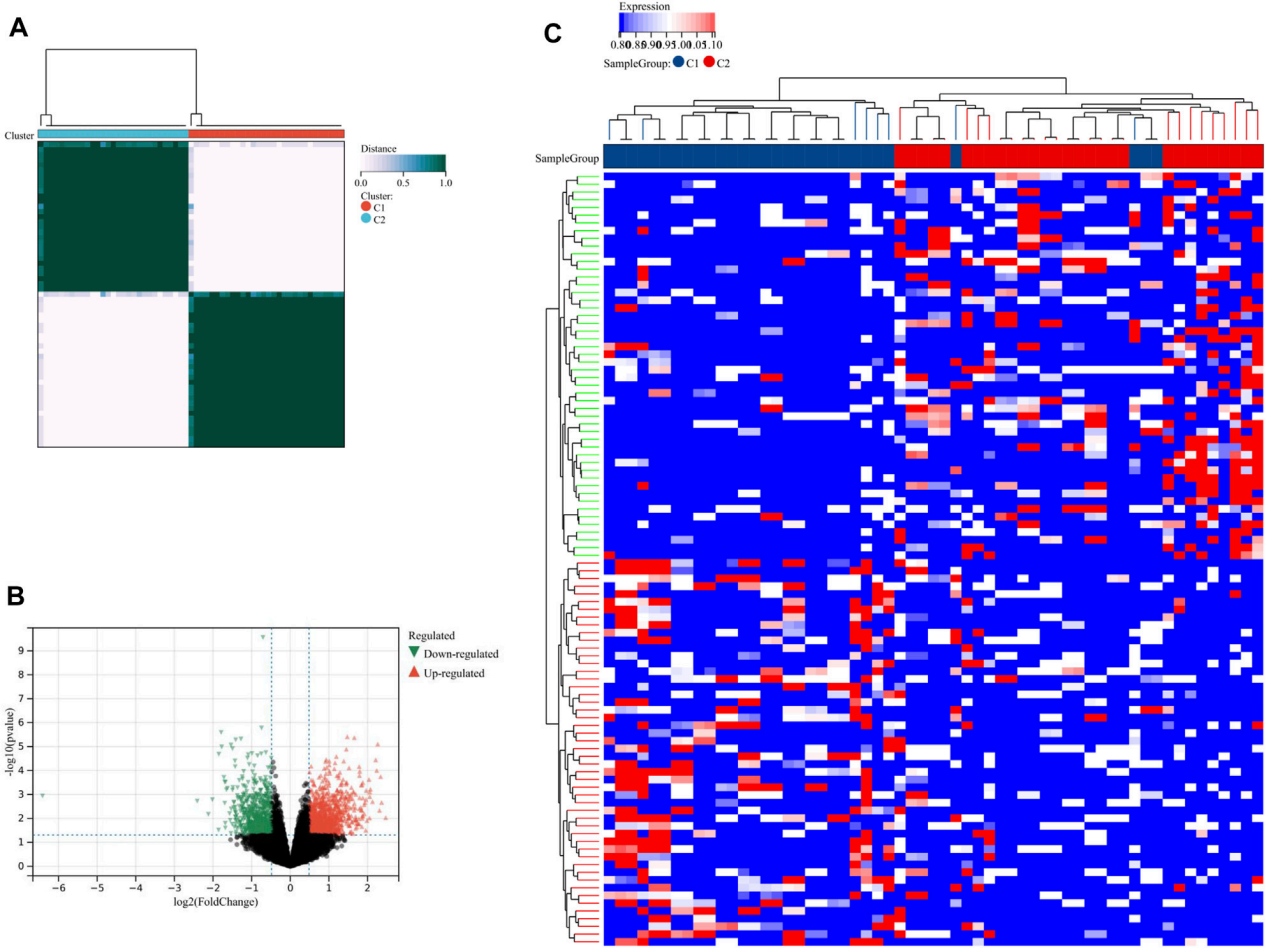
Identification of lysosome-related subtypes. **(A)** Unsupervised clustering analysis of LRs. The volcano plot **(B)** and heatmap **(C)** depicted the differential expression of genes between the two subtypes.

Using consensus clustering analysis, we were able to classify PE samples into two distinct molecular subtypes based on the LRs expression profiles. These subtypes were labeled as C1 (N = 30) and C2 (N = 29) (Figure 7A). To compare the two subtypes, a total of 1821 DEGs were identified between C1 and C2. Among these, 1060 DEGs were upregulated and 761 DEGs were downregulated in C2 (Figure 7B). The heatmap demonstrated that these DEGs were able to differentiate between the two lysosome patterns (Figure 7C).

### Functional enrichment analysis between two subgroups

**FIGURE 8:**
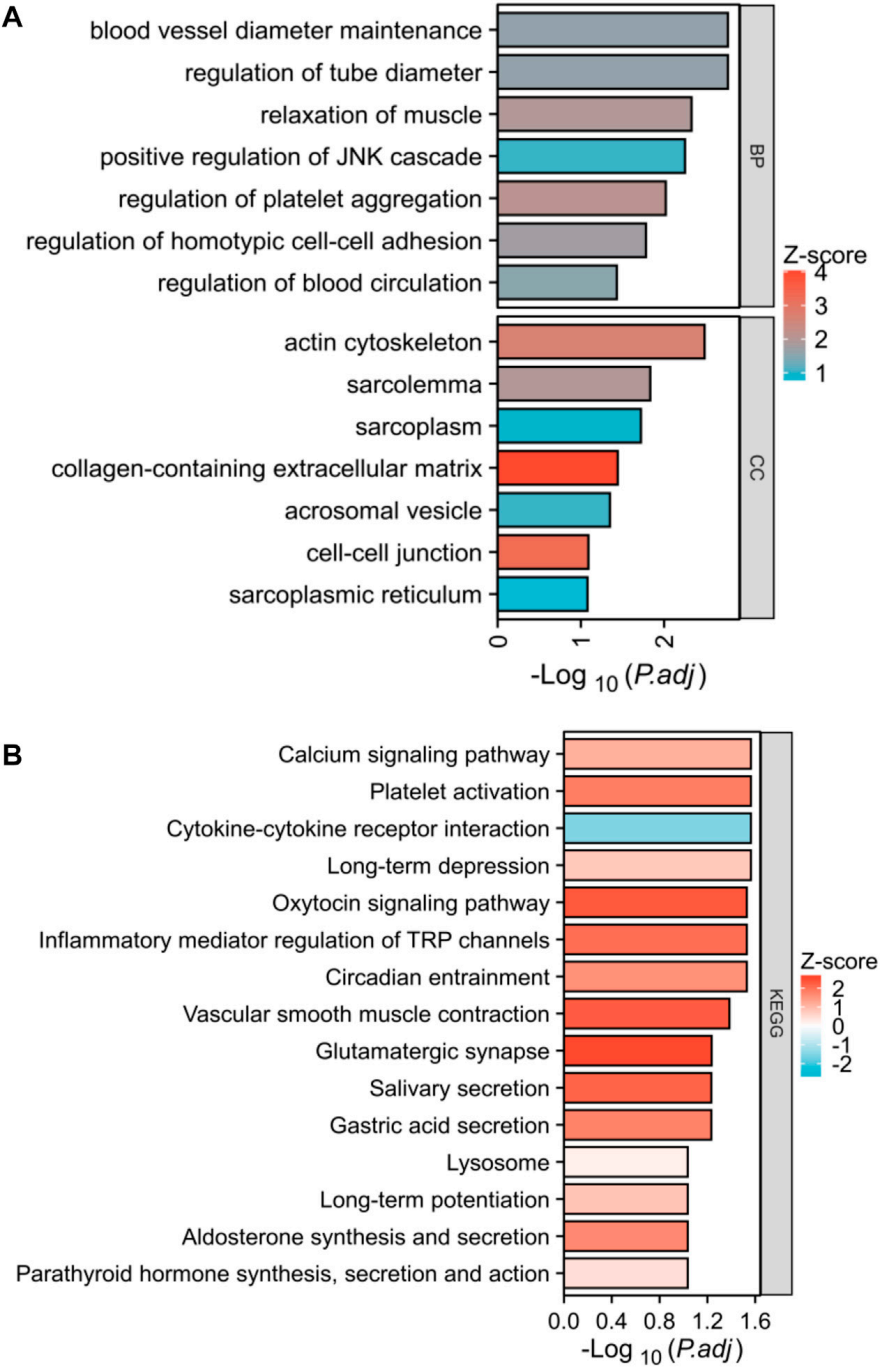
Biological functional enrichment analysis. **(A)** Functional enrichment in biological process and cellular component. **(B)** Histogram exhibited the results of the KEGG pathway analysis.

In this study, we analyzed the DEGs between two lysosome modalities in patients with PE and investigated their involvement in biologically significant functions. Our findings revealed that these DEGs were mainly enriched in BP of blood vessel diameter maintenance, regulation of tube diameter, positive regulation of JNK cascade, regulation of homotypic cell-cell adhesion, *etc.* The enriched CC of these genes were actin cytoskeleton, sarcolemma, cell-cell junction, sarcoplasmic reticulum, etc (Figure 8A). The enriched KEGG of these genes were the calcium signaling pathway, platelet activation, cytokine-cytokine receptor interaction, oxytocin signaling pathway, inflammatory mediator regulator of TRP channels, lysosome, etc (Figure 8B).

**FIGURE 9:**
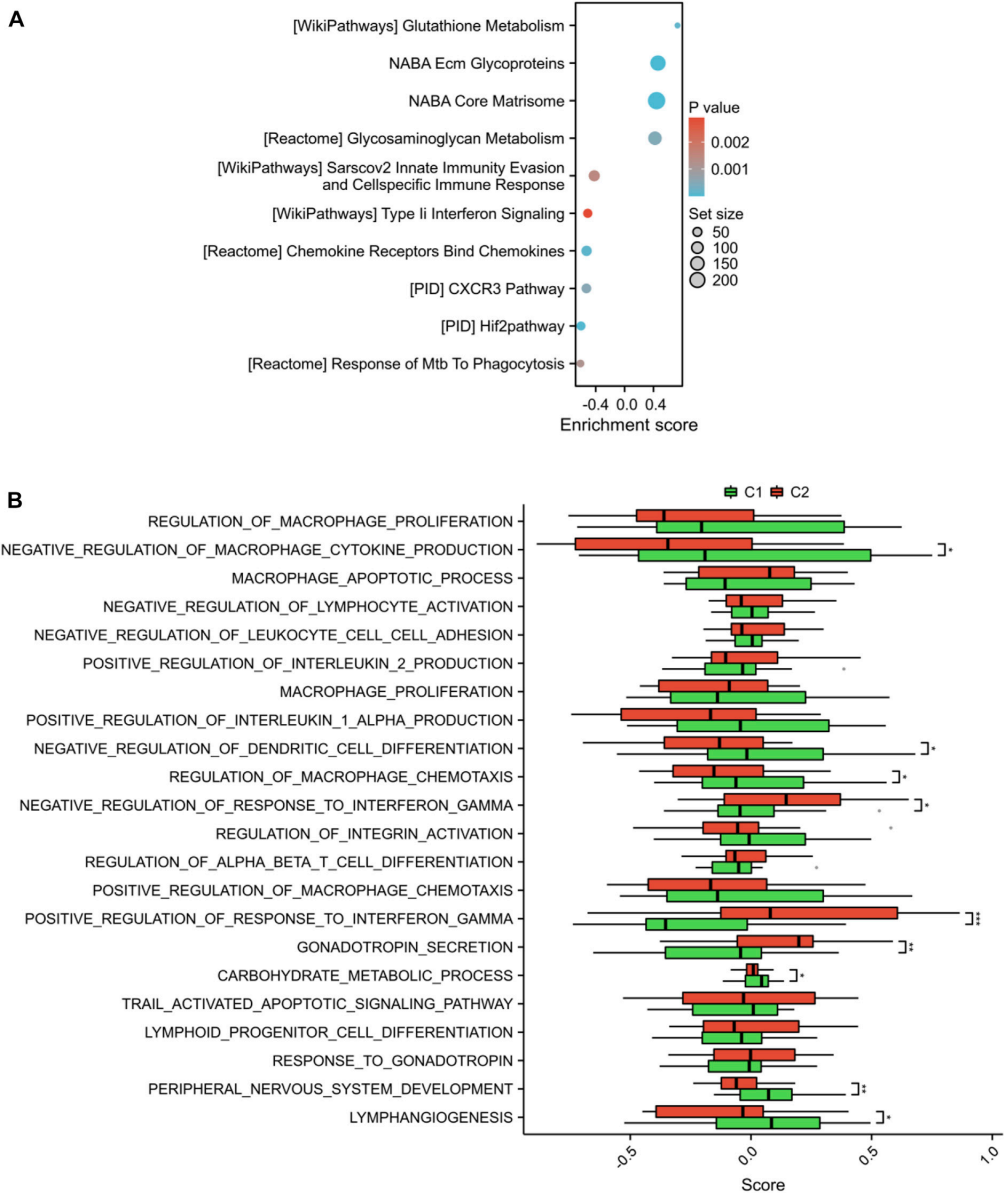
GSEA and GSVA between the two subtypes. **(A)** The bubble diagram exhibited the results of GSEA. **(B)** Histogram exhibited the results of GSVA. *p < 0.05, **p < 0.01, ***p < 0.001.

In addition, we performed GSEA analysis on all genes linked to two distinct lysosome patterns and observed notable variations in pathways, particularly in glutathione metabolism, NABA Ecm glycoproteins, glycosaminoglycan metabolism, sarscov2 innate immunity evasion and cell-specific immune response, type interferon signaling, chemokine receptors bind chemokines, CXCR3 pathway, etc (Figure 9A). Furthermore, GSVA results indicated that negative regulation of macrophage cytokine production, negative regulation of dendritic cell differentiation, regulation of macrophage chemotaxis, peripheral nervous system development, and lymphangiogenesis were upregulated in the C1 subtype, while positive regulation of response to interferon-gamma was downregulated in the C1 subtype (Figure 9B).

### Analysis of immune cell infiltration between the two subtypes

**FIGURE 10:**
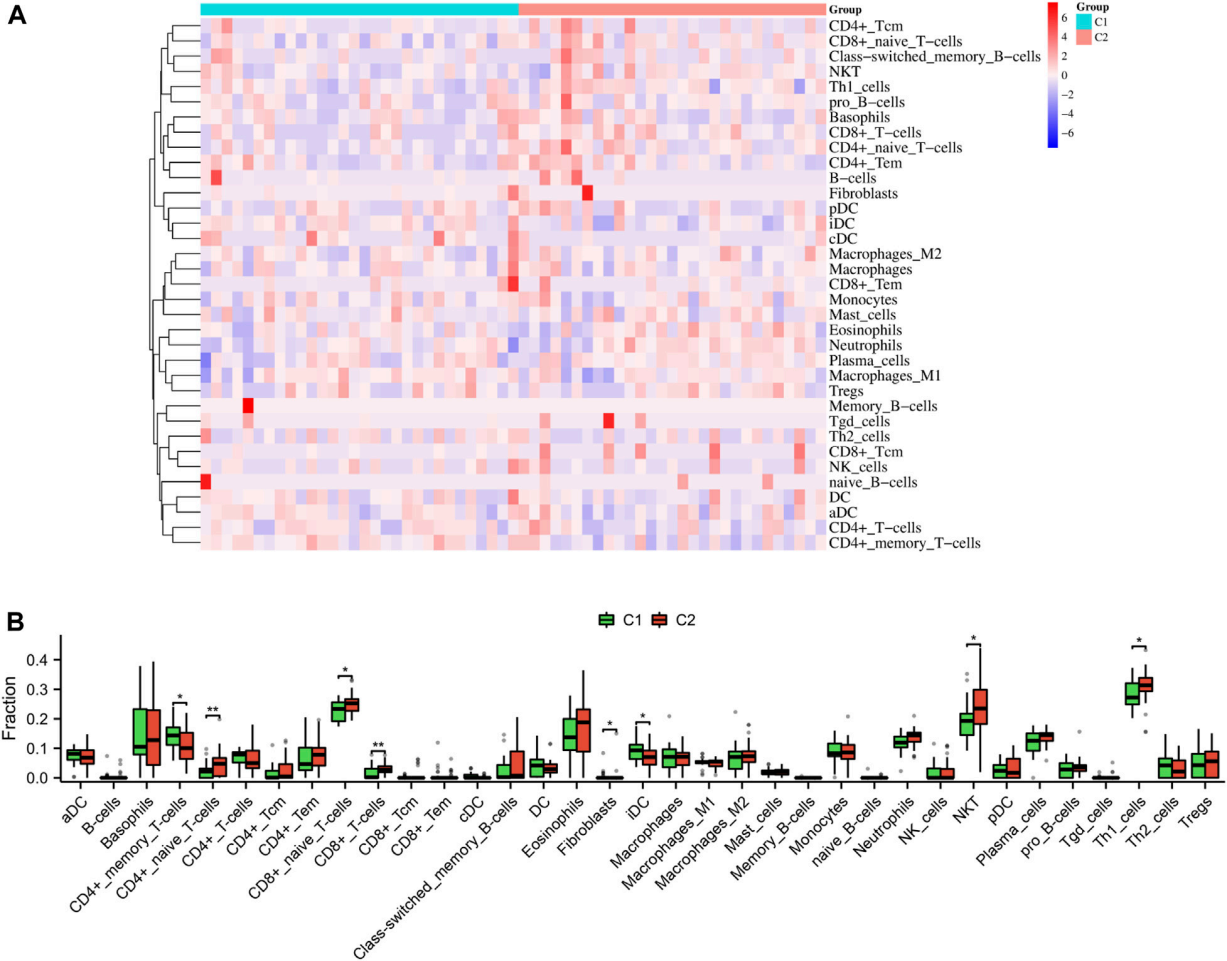
Immune characteristics between the two subtypes. Heatmap **(A)** and boxplot **(B)** depicted the differential infiltration of the immune cells.

As shown in Figures 10A, B, our findings that the proportion of CD4^+^ memory T cells (*p* < 0.05) and iDC (*p* < 0.05) was notably greater in the C1 subtype compared to the C2 subtype; however, the proportion of CD4^+^ naïve T cells (*p* < 0.01), CD8^+^ naïve T cells (*p* < 0.05), CD8^+^ T cells (*p* < 0.01), NKT (*p* < 0.05), and Th1 cells (*p* < 0.05) was notably lower in the C1 subtype compared to the C2 subtype.

## Discussion

Lysosomes are organelles responsible for digestion in both the autophagic and endocytic pathways. By increasing the activity of lysosomal enzymes, it may be possible to clear pathological waste from cells (Saftig and Haas, 2016). Lysosomal dysfunction has been demonstrated in the pathology of malignant tumors, pancreatitis, neurodegenerative diseases, and atherosclerosis (Zhang et al., 2021). Recent evidence suggested that lysosome is a significant factor in the onset and progression of PE (Nakashima et al., 2020; Ermini et al., 2021). As a result, biomarkers related to lysosomes could serve as valuable diagnostic tools and therapeutic targets for PE. Our study extracted the placental transcriptome from the GEO database of PE patients. Analysis of gene expression revealed 16 differentially expressed LRs in the PE group compared to the HC group. Subsequently, logistic regression analysis was performed on all the LRs, resulting in the identification of the key genes *GNPTG* and *CTSC*. A diagnostic model was constructed using these genes to predict the occurrence of PE.

The *GNPTG* gene is responsible for encoding the alpha and beta subunits, as well as the gamma subunit of the UDPGlcNAc-1-phosphotransferase enzyme, which is crucial for the correct transportation of lysosomal acid hydrolases (Leroy et al., 2014). Mucolipidosis types II and III are severe forms of autosomal recessive lysosomal storage diseases that can be caused by mutations in the *GNPTG* gene (Chen et al., 2015; Pasumarthi et al., 2020). A study of individuals with stuttering from Pakistan and North America revealed multiple mutations in the *GNPTG* gene associated with lysosomal enzyme targeting pathways (Kang et al., 2010). *CTSC* is a lysosomal cysteine proteinase that belongs to the papain superfamily (Diao et al., 2022). It is expressed in various inflammatory cells in mammals, such as epithelial cells, natural killer cells, and cytotoxic T cells (Diao et al., 2022). Deficiency of *CTSC* in mammals leads to the development of autoimmune diseases such as periodontitis, Halm-Munk syndrome, and Papillon-Lefevre syndrome (Hart et al., 2000; Nakano et al., 2001; Loos et al., 2005). Additionally, it plays a crucial role in the development and progression of tumors (Xiao et al., 2021; Cheng et al., 2022; Dang et al., 2023). Our study is the first to report on the potential of *GNPTG* and *CTSC* as diagnostic biomarkers for PE progression. As shown in GSE60438 dataset (Figure 3C) and in-house experiments (Figure 3E), the *GNPTG* gene was significantly upregulated in the PE group compared to the HC group, while *CTSC* was significantly downregulated in the PE group (*p* < 0.05). Our findings suggested that *GNPTG* and *CTSC* genes may serve as effective diagnostic biomarkers for PE patients.

According to our research, *GNPTG* and *CTSC* genes be correlated with immune cell infiltrations during the development of PE. PE is a condition where immune cells, including regulatory T cells, macrophages, natural killer cells, and neutrophils, play a significant role in its pathology (Aneman et al., 2020; Deer et al., 2023). Our study revealed that patients with PE can be classified into two distinct subgroups based on unsupervised clustering analysis, with varying levels of immune cell infiltration. Notably, in the C2 subgroup, there was an upregulation of CD4^+^ naive T cells, CD8^+^ naïve T cells, CD8^+^ T cells, Th1 cells, and natural killer T cells (NKT), whereas CD4+memory T cells and iDC were downregulated. Consistent with our results, a previous study has revealed that NKT cells may be regulators of the Th1/Th2 balance at the maternal-fetal interface (Tsuda et al., 2001). NKT cells secrete both IL-4 and IFN-γ cytokines, which are believed to play a role in regulating the balance between Th1 and Th2 cells within the endometrial tissue (Chen and Paul, 1997). In addition, NKT cells play an important role in the pathogenesis of PE (Hashemi et al., 2017). At the feto-maternal interface, CD8^+^ T cells play a critical role in immune tolerance. Severe PE is distinguished by changes in the expression of cytotoxic CD8^+^ T cells in the maternal peripheral blood and decidua, indicating their involvement in the feto-maternal immune tolerance and pathophysiology of PE (Soljic et al., 2021). Moreover, modifying the expression of certain miRNAs associated with T cells could serve as a promising avenue for the treatment of PE (Zolfaghari et al., 2021). In our study, a total of 1821 genes showed significant differences in expression between the two lysosome-related subgroups. These genes were enriched in the cytokine-cytokine receptor interaction, inflammatory mediator regulator of TRP channels, lysosome, and regulation of macrophage chemotaxis in further functional enrichment analyses including GO, KEGG, GSEA, and GSVA. These findings indicated that the progression of PE is influenced by pathways associated with the immune system and inflammation, which is consistent with previous studies (Zhao et al., 2021; Deer et al., 2023).

## Conclusion

Overall, *GNPTG* and *CTSC* were identified as diagnostic markers for PE patients. We have developed a diagnostic model for patients with PE that has shown promising diagnostic value. Our analysis of 16 LRs has revealed significant differences in the immune microenvironment between C1 and C2 subtypes. Our findings could facilitate the identification of early diagnostic indicators and the development of effective immunotherapy approaches against PE.

## Data Availability

The original contributions presented in the study are included in the article/Supplementary Material, further inquiries can be directed to the corresponding author.

## References

[B1] Aneman I. Pienaar D. Suvakov S. Simic T. P. Garovic V. D. McClements L. (2020). Mechanisms of key innate immune cells in early- and late-onset preeclampsia. Front. Immunol. 11, 1864. 10.3389/fimmu.2020.01864 33013837 PMC7462000

[B2] Aran D. (2020). Cell-type enrichment analysis of bulk transcriptomes using xCell. Methods Mol. Biol. Clift. N.J.) 2120, 263–276. 10.1007/978-1-0716-0327-7_19 32124326

[B3] Byrne M. Agerbo E. Bennedsen B. Eaton W. W. Mortensen P. B. (2007). Obstetric conditions and risk of first admission with schizophrenia: A Danish national register based study. Schizophrenia Res. 97 (1-3), 51–59. 10.1016/j.schres.2007.07.018 17764905

[B4] Chen H. Paul W. E. (1997). Cultured NK1.1+ CD4+ T cells produce large amounts of IL-4 and IFN-gamma upon activation by anti-CD3 or CD1. J. Immunol. 159 (5), 2240–2249. (Baltimore, Md, 1950). 10.4049/jimmunol.159.5.2240 9278312

[B5] Chen H. Xu J. Zhou Y. Gao Y. Wang G. Xia J. (2015). Association study of stuttering candidate genes GNPTAB, GNPTG and NAGPA with dyslexia in Chinese population. BMC Genet. 16, 7. 10.1186/s12863-015-0172-5 25643770 PMC4342093

[B6] Cheng X. Ren Z. Liu Z. Sun X. Qian R. Cao C. (2022). Cysteine cathepsin C: A novel potential biomarker for the diagnosis and prognosis of glioma. Cancer Cell. Int. 22 (1), 53. 10.1186/s12935-021-02417-6 35109832 PMC8812029

[B7] Dang Y. Z. Chen X. J. Yu J. Zhao S. H. Cao X. M. Wang Q. (2023). Cathepsin C promotes colorectal cancer metastasis by regulating immune escape through upregulating CSF1. Neoplasma 70 (1), 123–135. 10.4149/neo_2023_220726N757 36916928

[B8] Deer E. Herrock O. Campbell N. Cornelius D. Fitzgerald S. Amaral L. M. (2023). The role of immune cells and mediators in preeclampsia. Nat. Rev. Nephrol. 19 (4), 257–270. 10.1038/s41581-022-00670-0 36635411 PMC10038936

[B9] Diao Q. Du H. Zhao N. Wu Y. Du X. Sun Y. (2022). Cathepsin C (CTSC) contributes to the antibacterial immunity in golden pompano (Trachinotus ovatus). Fish shellfish Immunol. 128, 316–326. 10.1016/j.fsi.2022.07.078 35952999

[B10] Ermini L. Farrell A. Alahari S. Ausman J. Park C. Sallais J. (2021). Ceramide-induced lysosomal biogenesis and exocytosis in early-onset preeclampsia promotes exosomal release of SMPD1 causing endothelial dysfunction. Front. Cell. Dev. Biol. 9, 652651. 10.3389/fcell.2021.652651 34017832 PMC8130675

[B11] Griffith M. I. Mann J. R. McDermott S. (2011). The risk of intellectual disability in children born to mothers with preeclampsia or eclampsia with partial mediation by low birth weight. Hypertens. pregnancy 30 (1), 108–115. 10.3109/10641955.2010.507837 20846048

[B12] Hart T. C. Hart P. S. Michalec M. D. Zhang Y. Firatli E. Van Dyke T. E. (2000). Haim-Munk syndrome and Papillon-Lefèvre syndrome are allelic mutations in cathepsin C. J. Med. Genet. 37 (2), 88–94. 10.1136/jmg.37.2.88 10662807 PMC1734521

[B13] Hashemi V. Dolati S. Hosseini A. Gharibi T. Danaii S. Yousefi M. (2017). Natural killer T cells in Preeclampsia: An updated review. Biomed. Pharmacother. = Biomedecine Pharmacother. 95, 412–418. 10.1016/j.biopha.2017.08.077 28863381

[B14] Iasonos A. Schrag D. Raj G. V. Panageas K. S. (2008). How to build and interpret a nomogram for cancer prognosis. J. Clin. Oncol. official J. Am. Soc. Clin. Oncol. 26 (8), 1364–1370. 10.1200/JCO.2007.12.9791 18323559

[B15] Irgens H. U. Reisaeter L. Irgens L. M. Lie R. T. (2001). Long term mortality of mothers and fathers after pre-eclampsia: Population based cohort study. BMJ Clin. Res. ed.) 323 (7323), 1213–1217. 10.1136/bmj.323.7323.1213 PMC59993 11719411

[B16] Kang C. Riazuddin S. Mundorff J. Krasnewich D. Friedman P. Mullikin J. C. (2010). Mutations in the lysosomal enzyme-targeting pathway and persistent stuttering. N. Engl. J. Med. 362 (8), 677–685. 10.1056/NEJMoa0902630 20147709 PMC2936507

[B17] Kim Y. R. Jung I. Park G. Chang S. W. Cho H. Y. (2021). First-trimester screening for early preeclampsia risk using maternal characteristics and estimated placental volume. J. maternal-fetal neonatal Med. 34 (7), 1155–1160. the official journal of the European Association of Perinatal Medicine, the Federation of Asia and Oceania Perinatal Societies, the International Society of Perinatal Obstet. 10.1080/14767058.2019.1628207 31220966

[B18] Leroy J. G. Sillence D. Wood T. Barnes J. Lebel R. R. Friez M. J. (2014). A novel intermediate mucolipidosis II/IIIαβ caused by GNPTAB mutation in the cytosolic N-terminal domain. Eur. J. Hum. Genet. EJHG 22 (5), 594–601. 10.1038/ejhg.2013.207 24045841 PMC3992569

[B19] Loos B. G. John R. P. Laine M. L. (2005). Identification of genetic risk factors for periodontitis and possible mechanisms of action. J. Clin. periodontology 32 (6), 159–179. 10.1111/j.1600-051X.2005.00806.x 16128836

[B20] Ma'ayeh M. Costantine M. M. (2020). Prevention of preeclampsia. Seminars fetal & neonatal Med. 25 (5), 101123. 10.1016/j.siny.2020.101123 PMC8236336 32513597

[B21] Mahapatra K. K. Mishra S. R. Behera B. P. Patil S. Gewirtz D. A. Bhutia S. K. (2021). The lysosome as an imperative regulator of autophagy and cell death. Cell. Mol. Life Sci. 78 (23), 7435–7449. 10.1007/s00018-021-03988-3 34716768 PMC11071813

[B22] Mol B. W. J. Roberts C. T. Thangaratinam S. Magee L. A. de Groot C. J. M. Hofmeyr G. J. (2016). Pre-eclampsia. Lancet (London, Engl. 387 (10022), 999–1011. 10.1016/S0140-6736(15)00070-7 26342729

[B23] Nakano A. Nomura K. Nakano H. Ono Y. LaForgia S. Pulkkinen L. (2001). Papillon-lefèvre syndrome: Mutations and polymorphisms in the cathepsin C gene. J. investigative dermatology 116 (2), 339–343. 10.1046/j.1523-1747.2001.01244.x 11180012

[B24] Nakashima A. Cheng S. B. Ikawa M. Yoshimori T. Huber W. J. Menon R. (2020). Evidence for lysosomal biogenesis proteome defect and impaired autophagy in preeclampsia. Autophagy 16 (10), 1771–1785. 10.1080/15548627.2019.1707494 31856641 PMC8386603

[B25] Pasumarthi D. Gupta N. Sheth J. Jain S. Rungsung I. Kabra M. (2020). Identification and characterization of 30 novel pathogenic variations in 69 unrelated Indian patients with Mucolipidosis Type II and Type III. J. Hum. Genet. 65 (11), 971–984. 10.1038/s10038-020-0797-8 32651481

[B26] Radulovic M. Schink K. O. Wenzel E. M. Nähse V. Bongiovanni A. Lafont F. (2018). ESCRT-mediated lysosome repair precedes lysophagy and promotes cell survival. EMBO J. 37 (21), e99753. 10.15252/embj.201899753 30314966 PMC6213280

[B27] Ramos J. G. L. Sass N. Costa S. H. M. (2017). Preeclampsia. Rev. Bras. Ginecol. Obstet. Rev. Fed. Bras. das Soc. Ginecol. Obstet. 39 (9), 496–512. 10.1055/s-0037-1604471 PMC10309474 28793357

[B28] Saftig P. Haas A. (2016). Turn up the lysosome. Nat. Cell. Biol. 18 (10), 1025–1027. 10.1038/ncb3409 27684505

[B29] Saito S. Nakashima A. (2014). A review of the mechanism for poor placentation in early-onset preeclampsia: The role of autophagy in trophoblast invasion and vascular remodeling. J. reproductive Immunol. 101-102, 80–88. 10.1016/j.jri.2013.06.002 23969229

[B30] Skjaerven R. Wilcox A. J. Klungsøyr K. Irgens L. M. Vikse B. E. Vatten L. J. (2012). Cardiovascular mortality after pre-eclampsia in one child mothers: Prospective, population based cohort study. BMJ Clin. Res. ed.) 345, e7677. 10.1136/bmj.e7677 PMC3508198 23186909

[B31] Soljic V. Barbaric M. Vukoja M. Curlin M. Orlovic Vlaho M. Cerni Obrdalj E. (2021). Decreased expression of cytotoxic proteins in decidual CD8(+) T cells in preeclampsia. Biology 10 (10), 1037. 10.3390/biology10101037 34681139 PMC8533461

[B32] Taysi S. Tascan A. S. Ugur M. G. Demir M. (2019). Radicals, oxidative/nitrosative stress and preeclampsia. Mini Rev. Med. Chem. 19 (3), 178–193. 10.2174/1389557518666181015151350 30324879

[B33] Travaglino A. Raffone A. Saccone G. Migliorini S. Maruotti G. M. Esposito G. (2019). Placental morphology, apoptosis, angiogenesis and epithelial mechanisms in early-onset preeclampsia. Eur. J. obstetrics, Gynecol. reproductive Biol. 234, 200–206. 10.1016/j.ejogrb.2018.12.039 30721786

[B34] Tsuda H. Sakai M. Michimata T. Tanebe K. Hayakawa S. Saito S. (2001). Characterization of NKT cells in human peripheral blood and decidual lymphocytes. Am. J. reproductive Immunol. 45 (5), 295–302. (New York, N.Y, 1989). 10.1111/j.8755-8920.2001.450505.x 11432404

[B35] Ursini G. Punzi G. Chen Q. Marenco S. Robinson J. F. Porcelli A. (2018). Convergence of placenta biology and genetic risk for schizophrenia. Nat. Med. 24 (6), 792–801. 10.1038/s41591-018-0021-y 29808008

[B36] van der Merwe J. L. Hall D. R. Wright C. Schubert P. Grové D. (2010). Are early and late preeclampsia distinct subclasses of the disease-what does the placenta reveal? Hypertens. pregnancy 29 (4), 457–467. 10.3109/10641950903572282 20701467

[B37] Visser N. van Rijn B. B. Rijkers G. T. Franx A. Bruinse H. W. (2007). Inflammatory changes in preeclampsia: Current understanding of the maternal innate and adaptive immune response. Obstetrical Gynecol. Surv. 62 (3), 191–201. 10.1097/01.ogx.0000256779.06275.c4 17306041

[B38] Walker C. K. Krakowiak P. Baker A. Hansen R. L. Ozonoff S. Hertz-Picciotto I. (2015). Preeclampsia, placental insufficiency, and autism spectrum disorder or developmental delay. JAMA Pediatr. 169 (2), 154–162. 10.1001/jamapediatrics.2014.2645 25485869 PMC4416484

[B39] Wang F. Gómez-Sintes R. Boya P. (2018). Lysosomal membrane permeabilization and cell death. Traffic (Copenhagen, Den. 19 (12), 918–931. 10.1111/tra.12613 30125440

[B40] Wang Y. Li B. Zhao Y. (2022). Inflammation in preeclampsia: Genetic biomarkers, mechanisms, and therapeutic strategies. Front. Immunol. 13, 883404. 10.3389/fimmu.2022.883404 35880174 PMC9307876

[B41] Watts C. (2022). Lysosomes and lysosome-related organelles in immune responses. FEBS open bio 12 (4), 678–693. 10.1002/2211-5463.13388 PMC8972042 35220694

[B42] Wu C. S. Sun Y. Vestergaard M. Christensen J. Ness R. B. Haggerty C. L. (2008). Preeclampsia and risk for epilepsy in offspring. Pediatrics 122 (5), 1072–1078. 10.1542/peds.2007-3666 18977989

[B43] Xiao Y. Cong M. Li J. He D. Wu Q. Tian P. (2021). Cathepsin C promotes breast cancer lung metastasis by modulating neutrophil infiltration and neutrophil extracellular trap formation. Cancer Cell. 39 (3), 423–437.e7. 10.1016/j.ccell.2020.12.012 33450198

[B44] Yang C. Wang X. (2021). Lysosome biogenesis: Regulation and functions. J. Cell. Biol. 220 (6), e202102001. 10.1083/jcb.202102001 33950241 PMC8105738

[B45] Zhang J. Klebanoff M. A. Roberts J. M. (2001). Prediction of adverse outcomes by common definitions of hypertension in pregnancy. Obstetrics Gynecol. 97 (2), 261–267. 10.1016/s0029-7844(00)01125-x 11165592

[B46] Zhang Z. Yue P. Lu T. Wang Y. Wei Y. Wei X. (2021). Role of lysosomes in physiological activities, diseases, and therapy. J. Hematol. Oncol. 14 (1), 79. 10.1186/s13045-021-01087-1 33990205 PMC8120021

[B47] Zhao X. Chen S. Zhao C. Xia F. (2021). Maternal immune system and state of inflammation dictate the fate and severity of disease in preeclampsia. J. Immunol. Res. 2021, 1–10. 10.1155/2021/9947884 PMC8203389 34195300

[B48] Zolfaghari M. A. Arefnezhad R. Parhizkar F. Hejazi M. S. Motavalli Khiavi F. Mahmoodpoor A. (2021). T lymphocytes and preeclampsia: The potential role of T-cell subsets and related MicroRNAs in the pathogenesis of preeclampsia. Am. J. reproductive Immunol. 86 (5), e13475. (New York 1989). 10.1111/aji.13475 34043850

